# Ruptur des vorderen Kreuzbands: Was ist bei der Rekonstruktion zu beachten?

**DOI:** 10.1007/s00113-025-01551-4

**Published:** 2025-03-19

**Authors:** Christian Fink, Andrea Marchetti, Tobias Schwäblein, Mirco Herbort

**Affiliations:** 1 Gelenkpunkt – Sport und Gelenkchirurgie, Olympiastraße 39, 6020 Innsbruck, Österreich; 2 Research Unit für Sportmedizin des Bewegungsapparates und Verletzungsprävention, UMIT, Hall, Österreich; 3 Klinik für Orthopädie und Traumatologie, Universitätsklinikum, Triest, Italien; 4 Klinik für Unfall- und Wiederherstellungschirurgie, BG Klinikum Bergmannstrost, Halle (Saale), Deutschland; 5 OCM Klinik München, München, Deutschland

**Keywords:** Sportverletzungen, Gelenkinstabilität, Slope-Reduktion, Rotation, Begleitverletzungen, Athletic injuries, Joint instability, Slope reduction, Rotation, Concomitant injuries

## Abstract

Die Ruptur des vorderen Kreuzbands (VKB) ist eine häufige Sportverletzung. Nach ihrer Therapie kehren, trotz kontinuierlicher Verbesserung, nicht alle Patienten zu ihren präoperativen Aktivitäten zurück. Individualisierte Behandlungsansätze, basierend auf Transplantatwahl, Rekonstruktionstechnik und biomechanischen Faktoren wie tibialem Slope und Rotationsinstabilitäten, sind entscheidend. Autogene Transplantate weisen unterschiedliche Eigenschaften hinsichtlich Entnahmemorbidität, Einheilungsverhalten und Rerupturrisiko auf. Der individuelle Anspruch der Patienten sollte berücksichtigt werden. Operationstechnisch ist die korrekte Tunnelplatzierung anhand anatomischer Landmarken essenziell. Außerdem müssen Begleitinstabilitäten und Meniskusverletzungen adressiert werden. Im Fall einer Reruptur ist die exakte Ursachenanalyse notwendig. Der Behandlungserfolg ist wesentlich durch die präzise Diagnostik und Therapie sowohl des VKB-Risses als auch der verletzten Begleitstrukturen bestimmt.

## Abstract (en)

Rupture of the anterior cruciate ligament (ACL) is a common sports injury. Despite continuous improvements over the years, not all patients return to their preoperative activities after treatment of the ACL. Therefore, individualized treatment approaches based on transplant selection, reconstruction technique and biomechanical factors, such as the tibial slope and rotational instability are crucial. Autogenous transplants have different properties in terms of donor site morbidity, healing behavior and risk of rerupture. The individual needs of the patient should therefore be taken into consideration. In terms of the surgical technique, correct tunnel placement based on anatomical landmarks is essential. In addition, concomitant instabilities and meniscus injuries must be addressed. In the event of a rerupture, an exact analysis of the causes is necessary. Ultimately, the success of the treatment depends to a large extent on precise diagnostics and the treatment of both the ACL rupture and any injured accompanying structures.

## Lernziele

Nach der Lektüre dieses Beitrags

- wissen Sie, anatomische Besonderheiten bei der Rekonstruktion des vorderen Kreuzbands (VKB) zu berücksichtigen.
- können Sie grundlegende Aspekte bei der Transplantatwahl differenzieren.
- ziehen Sie die richtigen Schlüsse aus der Wichtigkeit peripherer Strukturen und ihrem Einfluss auf die Rotationsstabilität.
- können Sie Indikationen zur Slope-Reduktion benennen.
- können Sie die beschriebenen Modifikationen im eigenen Patientenklientel anwenden.

## Einführung

Die Ruptur des VKB ist eine der häufigsten **Sportverletzungen**

Sportverletzungen,

und ihre Häufigkeit nimmt mit einer jährlichen Inzidenz bis zu 68,6/100.000 Personenjahre auf allen Wettkampfebenen zu [1]. Obwohl die VKB-Rekonstruktion zu den häufigsten orthopädischen Eingriffen gehört, kommt es trotz erheblicher Fortschritte bei der chirurgischen Behandlung immer noch bei 5–20 % der Patienten nach der Operation zu einer erneuten Instabilität [2].

Während das Hauptaugenmerk häufig auf der Rekonstruktion der anatomischen Strukturen des VKB selbst liegt, ist die Beachtung der umgebenden Strukturen des Knies ebenfalls von entscheidender Bedeutung, um die Operationsergebnisse zu verbessern und die Ausfallraten zu verringern. Dies hat zu VKB-Rekonstruktionen, die auf stärker individuellen und auf quantitativen Parametern der **Rotationslaxität**

Rotationslaxität

des Knies basiert, geführt. Ziel ist es, eine möglichst anatomiegerechte Rekonstruktion des gerissenen VKB zu gewährleisten, indem das für den Patienten am besten geeignete Transplantat ausgewählt wird, eine **Bohrkanalanlage**

Bohrkanalanlage

anhand anatomischer Leitstrukturen gewählt wird und sekundäre Faktoren wie die posteriore Neigung des Tibiaplateaus („posteriorer tibialer Slope“), Meniskusstrukturen sowie mediale und laterale Bandstrukturen berücksichtigt werden.

## Transplantatwahl

Die Wahl des Transplantats hat sich als modifizierbarer extrinsischer Faktor erwiesen, der die Misserfolgsrate der VKB-Rekonstruktion beeinflussen kann. Bei der modernen VKB-Rekonstruktion ist eine individuelle Auswahl des Transplantats von entscheidender Bedeutung, da kein einziges Transplantat optimal für jeden Patienten geeignet ist. Da alle Transplantatoptionen Vor- und Nachteile haben, muss die Wahl auf die anatomische Situation, das Aktivitätsniveau, das Alter, die Sportart, frühere Operationen, Begleitverletzungen und Achsverhältnisse des jeweiligen Patienten abgestimmt werden.

Die erste Entscheidung muss zwischen **autogenen Transplantaten**

autogenen Transplantaten

und **allogenen Transplantaten**

allogenen Transplantaten

getroffen werden. Letztere weisen jedoch v. a. bei jüngeren und sportlicheren Patienten eine höhere Versager- und Komplikationsrate, sowie höhere Kosten auf. Als autogene Transplantate stehen Hamstring-Sehnen (HS), „bone-patellar tendon-bone“ (BPTB), Quadrizepssehne (QS) mit oder ohne Knochenblock und Peronaeus-Longus-Split-Transplantate (PLS) zur Verfügung, wobei der **„bone-patellar tendon-bone“**

**Abb. 1:**
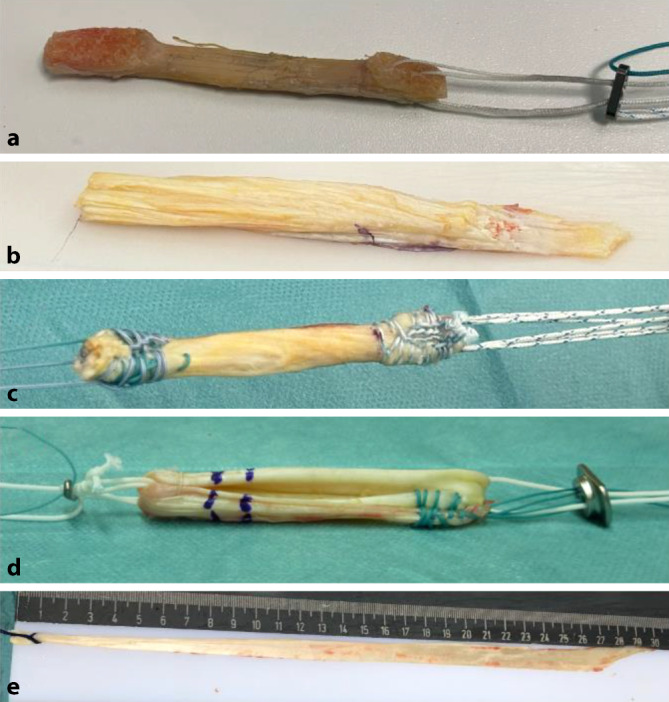
Sehnentransplantate zur Rekonstruktion des vorderen Kreuzbands (VKB). **a** Patellarsehne („bone-patellar tendon-bone“, *BPTB*), **b** Quadrizepssehne nach Entnahme mit Periostlappen, **c** Quadrizepssehne fertig armiert, **d** Semitendinosussehne, **e** Peronaeus-Longus-Sehne

„bone-patellar tendon-bone“

in der Vergangenheit insbesondere bei Hochleistungssportlern als „Goldstandard“ für die VKB-Rekonstruktion galt ([3]; Abb. 1).

Die Nachteile des BPTB bestehen darin, dass es bei Patienten mit offenen Wachstumsfugen nicht verwendet werden kann, und dass dieses Transplantat aufgrund des vorkommenden vorderen Knieschmerzes bei Personen, die häufig knien müssen, aufgrund der **Entnahmemorbidität**

Entnahmemorbidität

nicht geeignet ist. Die **Quadrizepssehne**

Quadrizepssehne

erfreut sich zunehmender Beliebtheit und ist eine weitere gute Option für Spitzensportler. Außerdem kann sie ohne den Knochenblock bei Patienten mit offenen Wachstumsfugen eingesetzt werden. Biomechanisch hat die QS im Vergleich zu den anderen Transplantaten bessere Struktureigenschaften (Bruchlast, Bruchdehnung und Elastizitätsmodul, [4]).

Während die subjektiven und objektiven Outcome Scores der Patienten und die postoperativen Stabilitätswerte bei den unterschiedlichen Transplantaten ähnlich zu sein scheinen [5, 6], konnte in einigen Studien sowohl bei Erwachsenen als auch besonders bei jugendlichen Patienten eine geringere Rerupturrate bei Rekonstruktionen der QS im Vergleich zu den HS-Rekonstruktionen aufgezeigt werden. Aufgrund der **minimalinvasiven Entnahmetechniken**

minimalinvasiven Entnahmetechniken

konnte zusätzlich bei QS-Transplantaten eine besonders geringe Entnahmemorbidität festgestellt werden [7].

Darüber hinaus bleibt bei der Verwendung von QS-Transplantaten der HS-Komplex erhalten; dieser fungiert als Synergist zum VKB bei der Begrenzung der anterioren tibialen Translation und als Agonist des Innenbandkomplexes bei der Begrenzung des Valgusmoments [8]. In Revisionsfällen, in denen ein Knochenblock erforderlich ist, kann die QS ebenfalls mit einem Knochenblock entnommen werden.

Bei Patienten mit mäßigen Aktivitätsanforderungen ist die die **Hamstring-Sehne**

Hamstring-Sehne

jedoch immer noch eine sehr gute Option.

## Chirurgische Anatomie

Die ideale VKB-Rekonstruktion besteht darin, „das VKB in seinen ursprünglichen Dimensionen, seiner einzelnen Faserausrichtung und seiner originären Insertionszone wiederherzustellen“, und erfordert ein tiefes Verständnis der Anatomie und Morphologie des Bandes [9]. Darüber hinaus spielt die genaue Positionierung des Transplantats eine entscheidende Rolle bei der Wiederherstellung der **Stabilität**

Stabilität

und **Kinematik**

Kinematik

des Kniegelenks und bei der Verhinderung eines Transplantat-Impingement und eines Transplantatversagens [10, 11, 12].

**Abb. 2:**
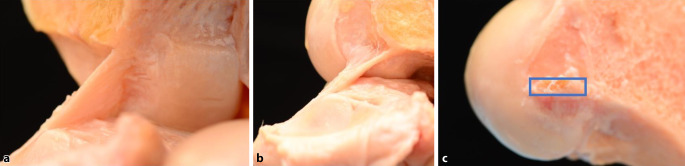
Flache „bandartige“ Struktur des vorderen Kreuzbands (VKB) **a** in Extension, **b** in 90° Flexion und korrespondierender rechteckiger femoraler Insertionspunkt (*Rechteck*, **c**)

Nach den Ergebnissen mehrerer aktueller Studien scheint das VKB, einschließlich seiner femoralen und tibialen Ansätze, eine flache „bandartige“ Struktur zu sein, bei der keine strukturelle Unterscheidung zwischen den beiden klassischen Bündeln feststellbar ist ([13, 14]; Abb. 2). Das native VKB ist etwa 11–16 mm breit und 2,5–3,4 mm dick, mit einer mittleren Querschnittsfläche von 56,6 mm^2^ und 39,8 mm^2^. Die **femorale Insertion**

femorale Insertion

hat eine dünne „direkte“ Insertion in Kontinuität mit der hinteren Kortikalis und erstreckt sich durch den lateralen interkondylären Kamm. Diese „direkte“ Insertion befindet sich in der Vertiefung zwischen dem lateralen interkondylären Kamm („resident’s ridge“) und 7–10 mm anterior des Gelenkknorpelrands. Darüber hinaus erstrecken sich fächerförmige **„indirekte“ Fasern**

„indirekte“ Fasern

in Richtung des hinteren Knorpelrands des Femurkondylus [14]. Der **tibiale Ansatz**

tibiale Ansatz

bildet eine halbmondförmige C‑, J‑ oder Cc-Form um das Vorderhorn des lateralen Meniskus [15].

**Abb. 3:**
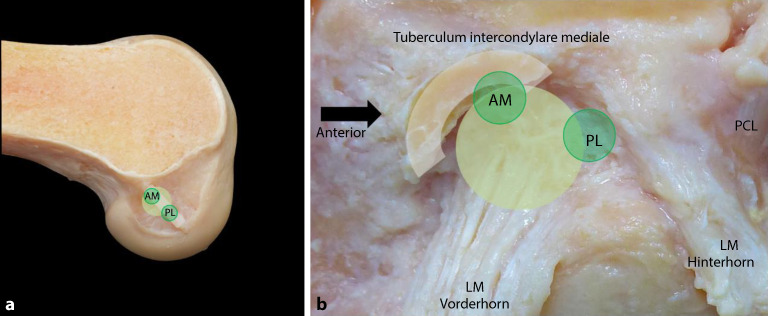
Anatomische Orientierung eines runden Bohrkanals femoral (**a**) und tibial (**b**) in Relation zum Vorderhorn des Außenmeniskus in *hellgrün*; *dunkelgrüne Punkte* als Insertionspunkte der vorherigen anteromedialen (*AM*) bzw. posterolateralen (*PL*) Bündel als Vergleich

Gemäß der biomechanischen Studie von Kawaguchi et al. sollte der **femorale Tunnel**

femorale Tunnel

in denen am stärksten belasteten Fasern des VKB platziert werden, was ungefähr der früheren „anteromedialen (AM-)Position“ entspricht. Ein an dieser Stelle platzierter runder Tunnel deckt der biomechanischen Studie folgend etwa 70–80 % der biomechanisch wichtigsten Fasern des VKB ab ([16]; Abb. 3). Alternativ kann ein rechteckiger oder ein Doppeltunnel, der zusätzlich noch mehr der wichtigen VKB-Fasern abdeckt, in diesem Bereich platziert werden [17].

**Abb. 4:**
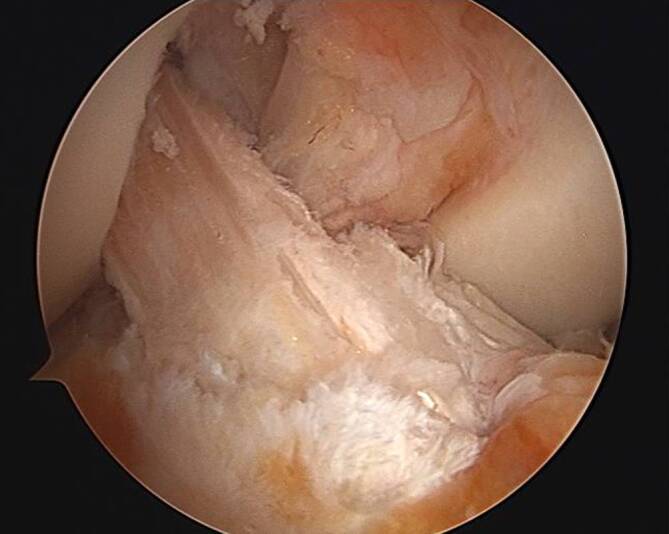
Arthroskopische Sicht auf ein korrekt rekonstruiertes vorderes Kreuzband mithilfe der Quadrizepssehne

In akuten Fällen kann der VKB-Stumpf eine gute Orientierungshilfe für die Platzierung des **tibialen Tunnels**

tibialen Tunnels

sein [18]. In chronischen Fällen, in denen die Reste möglicherweise nicht sichtbar sind, werden knöcherne Orientierungspunkte sowie Meniskusansatzstellen für die korrekte Tunnelplatzierung wichtiger. Für den femoralen Ansatz wird der Bereich superior durch eine Linie, die von der posterioren Kortikalis des Femurs nach anterior, posteroinferior durch den posterioren Gelenkrand und anterior durch die „resident’s ridge“ verläuft, begrenzt. Als arthroskopische Landmarke für das Schienbein bietet es sich an, den Tunnel medial zwischen der Mittellinie und der hinteren Begrenzung des Vorderhorns des Außenmeniskus zu platzieren (Abb. 4).

Beim Bohren des tibialen Tunnels sollte besonders darauf geachtet werden, dass der **Meniskuswurzelansatz**

Meniskuswurzelansatz

erhalten bleibt, um eine iatrogene Avulsion der vorderen oder hinteren Außenmeniskuswurzel zu vermeiden [19].

## Tibialer Slope

In der veterinärmedizinischen Literatur wurde ein Zusammenhang zwischen dem posterioren tibialen Slope und der VKB-Verletzung festgestellt. Dieser Erkenntnis folgend werden seit den 1980er-Jahren Slope-reduzierende Osteotomien zur Behandlung der anterioren Knielaxität bei Hunden durchgeführt [20]. In Übertragung dieser Konzepte auf menschliche Kniegelenke haben mehrere biomechanische Studien den Zusammenhang zwischen dem posterioren tibialen Slope (PTS) und **sagittaler Instabilität**

sagittaler Instabilität

untersucht.

Aus biomechanischer Sicht erzeugt ein erhöhter PTS bei einer axialen Druckbelastung des Knies eine nach anterior gerichtete Scherkraft, die eine anteriore tibiale Translation (ATT) verursacht [21, 22]. Da das VKB die ATT primär einschränkt, kann der PTS die auf das rekonstruierte VKB einwirkenden Kräfte relevant beeinflussen und zum Versagen des Transplantats führen [23].

Sowohl die mediale als auch die laterale posteriore Neigung des Tibiaplateaus ist mit einem erhöhten Risiko eines **Transplantatversagens**

Transplantatversagens

verbunden. Insbesondere eine steilere mediale PTS scheint für eine erhöhte statische und dynamische ATT verantwortlich zu sein [24], während eine höhere laterale Neigung des Tibiaplateaus v. a. mit einer erhöhten rotatorischen Instabilität verbunden ist [25].

Einige Autoren schlagen vor, dass eine **Osteotomie**

Osteotomie

zur Korrektur des Neigungswinkels für Patienten mit einem PTS ≥ 12° von Vorteil ist, da sie das Versagen des Transplantats minimieren kann [26, 27, 28]. In diesem Zusammenhang wurden verschiedene Techniken der anterioren schließenden Keilosteotomie („anterior closing wedge osteotomy“, ACWO) entwickelt, um die ATT zu reduzieren, indem eine Korrektur des erhöhten PTS während der primären oder Revisions-VKB-Rekonstruktion ermöglicht wird [29, 30].

Anhand ihrer Beziehung zur **Tuberositas tibiae**

Tuberositas tibiae

(TT) lassen sich die ACWO in 3 Untertypen einteilen: supratuberositär, transtuberositär und infratuberositär.

Die transtuberositäre ACWO wurde erstmals von Sonnery-Cottet et al. beschrieben; die Autoren führten die Osteotomie auf Höhe der TT durch. Die Osteotomie wurde mit 2 Klammern stabilisiert, während zur Fixierung der TT 2 Zugschrauben verwendet wurden [31, 32]. Nachteil einer supratuberositären ACWO ist, dass eine vorbestehende **Patella alta**

Patella alta

verstärkt und der VKB-Tunnel beeinträchtigt werden kann [33]. Im deutschen Sprachraum wird deshalb eher eine infratuberositäre ACWO bevorzugt. Wie von Hees und Petersen [34] sowie Ollivier et al. [35] beschrieben, kann eine ACWO unterhalb des TT viele Vorteile bieten. Ein Vorteil ist, dass sie bei Patienten mit Patella alta leicht durchgeführt werden kann, ohne die TT zu beeinträchtigen. Außerdem ermöglicht sie durch einen aufsteigenden Osteotomieschnitt einen relativ einfachen Schluss der Osteotomie. In Anbetracht der Tatsache, dass die Osteotomie weiter distal und mit einem schrägen Tibiaschnitt durchgeführt werden muss, erfordert diese Technik jedoch eine stabilere Fixierung (Plattenosteosynthese).

Zu den relativen Indikationen für die ACWO gehören fehlgeschlagene primäre VKB-Rekonstruktionen mit einem PTS ≥ 12°. Relative Kontraindikationen des Verfahrens sind massive **Hyperextensionsfähigkeit**

Hyperextensionsfähigkeit

des Knies (> 10°), Varus- und Valgusdeformität von mehr als 10°, hintere Kreuzbandinsuffizienz und schwere Arthrose des Knies (Grade 3 und 4 nach Kellgren-Lawrence; [36]).

In der Literatur werden vielversprechende Ergebnisse mit verschiedenen Techniken vorgestellt, aber die meisten dieser Studien sind durch kleine Stichproben und eine kurze Nachbeobachtungszeit begrenzt. Nichtsdestotrotz wurde berichtet, dass mit verschiedenen Techniken gute Ergebnisse in Bezug auf die Kniestabilität und die Rückkehr zu normalen Aktivitäten erzielt werden können [31, 37, 38, 39].

**Abb. 5:**
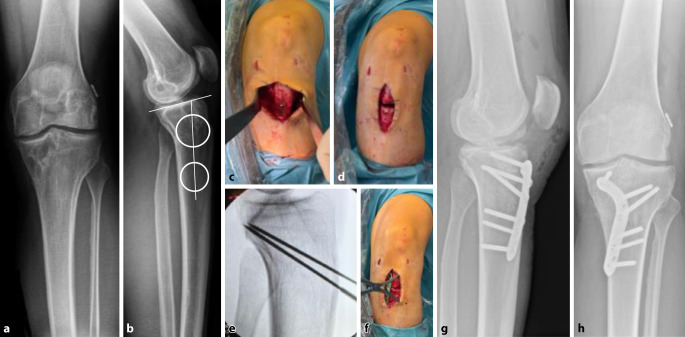
**a**, **b** Röntgenaufnahmen eines 30-jährigen Patienten mit Z. n. 2‑maliger Reruptur des vorderes Kreuzbands zeigen einen posterioren tibialen Slope von 16°. Zur Vorbereitung einer erneuten VKB-Rekonstruktion im Rahmen eines zweizeitigen Verfahrens wurde zunächst eine infratuberositär schließende Osteotomie (**c–f**), einschließlich einer Auffüllung der Bohrkanäle, durchgeführt. Hierbei wurde eine Reduktion des Slope um 6° erzielt (**g**, **h**)

Obwohl für die Durchführung einer ACWO während der primären VKB-Rekonstruktion oder der ersten VKB-Revision günstige Ergebnisse berichtet wurden, führen wir sie in der Mehrzahl der Fälle während der zweiten Revision bei Patienten mit einem PTS > 12 durch (Abb. 5). Allerdings ziehen wir eine Slope-Korrektur bei der primären VKB-Rekonstruktion in Betracht, wenn der Slope > 18° beträgt oder nach einem nichttraumatischen Versagen des Transplantats auf der kontralateralen Seite und einem Slope > 16°. Zur Beurteilung der Neigung wird daher empfohlen, auch bei der primären VKB-Rekonstruktion routinemäßig eine lange seitliche Röntgenaufnahme der Tibia anzufertigen. In einigen Berichten wird empfohlen, den PTS auf 4–6° zu korrigieren [40], jedoch ist insbesondere bei Patienten mit einer präoperativen Hyperextension oder einem erheblichen präoperativen Slope Vorsicht geboten. In diesen Fällen kann eine zu starke Korrektur der Neigung zu einer Verstärkung der Hyperextension führen.

### Merke

Eine standardisierte Slope-Reduktion auf einen generellen Zielwert sollte nicht durchgeführt werden.

## Rotationsinstabilitäten

Die Rotationsinstabilität des Knies ist einer der Hauptgründe für Misserfolge und schlechte klinische Ergebnisse nach einer VKB-Rekonstruktion. In der Vergangenheit lag der Schwerpunkt v. a. auf der anterolateralen Instabilität, während das mediale Kompartiment oft als „vernachlässigte Ecke“ oder „vernachlässigtes Band“ angesehen wurde, z. T. aufgrund der Überzeugung, dass das mediale Kompartiment des Knies ein enormes Heilungspotenzial besitzt und ein chirurgischer Eingriff nur selten erforderlich ist.

### Anterolaterale Instabilität

Bei der anterolateralen Rotationsinstabilität (ALRI) handelt es sich um eine kombinierte anteriore Translation und Innenrotation des Schienbeinkopfes, die auftritt, wenn die anterolateralen Strukturen des Knies (Tractus iliotibialis [ITB]/Kaplan-Fasern, anterolaterales Band [„anterolateral ligament“, ALL] und Kapsel) in Verbindung mit dem VKB verletzt werden (in 10,8–62,5 % der Fälle; [41]).

In der Tat fungiert das VKB in der Streckung als primärer Stabilisator der tibialen Innenrotation, während in der Beugung die anterolateralen Strukturen rekrutiert werden (das ITB näher an der Streckung, das ALL bei höherer Beugung) und als Hauptstabilisatoren der tibialen Innenrotation wirken. Daher wird eine VKB-Ruptur als Voraussetzung für die Entwicklung einer ALRI angesehen. Die ALRI kann sich auch nach einer gleichzeitigen Verletzung der anterolateralen Strukturen entwickeln [42]. Aus biomechanischer Sicht kann die ALRI außerdem durch die anatomische knöcherne Situation des Schienbeins verstärkt werden. Je steiler der Slope, desto größer dürfte die ALRI sein, wenn die anterolateralen Strukturen verletzt sind, da ein Oberschenkelknochen an einem steileren Slope dazu neigt, mehr nach hinten zu gleiten [43]. Zusätzlich führt das Vorhandensein einer **posterolateralen Tibiaplateaufraktur**

posterolateralen Tibiaplateaufraktur

(auch als „Bankart-Läsion des Kniegelenks“ bekannt), die mit einem Verlust der knöchernen Unterstützung für das Hinterhorn des lateralen Meniskus einhergeht, zu einer zusätzlichen Zunahme der anterioren tibialen Translation und der anterolateralen Rotationsinstabilität des VKB-defizienten Gelenks [44].

Es ist wichtig, eine ALRI zu erkennen, da sie unbehandelt für eingeschränkte Operationsergebnisse, ein mögliches Versagen der VKB-Rekonstruktion und sekundäre Verletzungen des Meniskus oder Knorpels sowie das frühzeitige Auftreten von **arthritischer Degeneration**

arthritischer Degeneration

verantwortlich sein kann.

Klinisch äußert sich die ALRI durch einen hochgradigen positiven Ausfall des **Pivot-Shift-Tests**

Pivot-Shift-Tests.

Röntgenaufnahmen können die **Segond-Fraktur**

Segond-Fraktur,

die als tibialer Abriss des anterolateralen Komplexes definiert ist, zeigen, und die MRT kann die Läsionen der anterolateralen Strukturen bei genauer Analyse meist gut darstellen.

Derzeit gibt es keine eindeutigen evidenzbasierten Indikationen für die Durchführung eines anterolateralen extraartikulären Eingriffs im Zusammenhang mit einer VKB-Rekonstruktion. Die Entscheidung basiert hauptsächlich auf der präoperativen klinischen Bewertung und den Patientenmerkmalen. In der klinischen Praxis werden Revisionsoperationen, hochgradige Pivot-Shift-Test-Befunde, generalisierte Hyperlaxität, Genu recurvatum oder Hyperextension > 10° und Patienten im Alter < 25 Jahren, die wieder pivotierende Sportarten ausüben, als Indikationen angegeben [45].

Es gibt auch keine eindeutige Überlegenheit einer ALL-Rekonstruktion gegenüber den verschiedenen Formen der anterolateralen Tenodese (laterale extraartikuläre Tenodese, LET).

### Anteromediale Instabilität

Slocum und Larson beschrieben 1968 erstmals die anteromediale Rotationsinstabilität (AMRI) als eine erhöhte Außenrotation der Tibia mit anteriorer Subluxation des medialen Tibiaplateaus [46].

Da eine kombinierte VKB- und „Medial-collateral-ligament“(MCL)-Verletzung die häufigste Bandverletzung des Knies darstellt, sollte bei der Untersuchung eines VKB-verletzten Kniegelenks auf eine **mediale Begleitverletzung**

mediale Begleitverletzung

untersucht werden. Klinische Studien haben bestätigt, dass eine unbehandelte mediale Laxität mit einem 13- bis 17fach erhöhten Risiko eines VKB-Transplantat-Versagens verbunden ist [47, 48].

Das oberflächliche MCL („superficial [s]MCL“) ist der wichtigste Stabilisator für die Valgusbelastung im gesamten Bewegungsbereich und für die Außenrotation der Tibia in Flexion, während das hintere Schrägband („posterior oblique ligament“, POL) in voller Streckung einen wichtigen Stabilisator für die Valgusrotation und die Tibiainnendrehung darstellt. Darüber hinaus ist das tiefe MCL („deep [d]MCL“) der primäre Stabilisator für die tibiale Außenrotation von 0°- bis 30°-Flexion [49] und hat, wie Willinger et al. feststellten, analog eine vergleichbare Funktion wie das ALL auf der lateralen Seite [50].

Die klinische Bewertung der medialen und anteromedialen Knieinstabilität umfasst spezifische Tests, mit denen sowohl die Laxität als auch die Rotationsstabilität beurteilt werden sollen. Der **Valgusbelastungstest**

Valgusbelastungstest

wird üblicherweise zur Beurteilung der medialen Laxität verwendet; insbesondere in Fällen einer sMCL-Beteiligung ist die Valgusbelastung bei etwa 30° Flexion positiv. Während der **Dial-Test**

Dial-Test

(maximale Außenrotation des Unterschenkels in 60°-Beugung) in erster Linie dazu dient, eine posterolaterale Rotationsinstabilität festzustellen, kann er auch eine übermäßige anteromediale Rotation und eine Ruptur des dMCL aufzeigen.

In chronischen Fällen können **Belastungsröntgenaufnahmen**

Belastungsröntgenaufnahmen

in 30°-Beugung zur Beurteilung der Valgusinstabilität herangezogen werden: hierbei dient das kontralaterale unverletzte Knie als Referenz für die Messung der **medialen Gelenkspaltverbreiterung**

medialen Gelenkspaltverbreiterung.

#### Merke

Eine dezidierte klinische Untersuchung ist entscheidend, um verletzte Strukturen zu identifizieren.

In akuten Fällen helfen die MRT-Aufnahmen beim Nachweis einer Bandläsion, indem sie direkt die **Weichteilrisse**

Weichteilrisse

oder indirekt die **Knochenprellungen**

Knochenprellungen

darstellen; darüber hinaus kann auch der Ultraschall zur Beurteilung von MCL-Verletzungen und zur Messung der medialen Gelenkspaltweite nützlich sein. Eine Rotationsinstabilität kann jedoch in Belastungsröntgenaufnahmen und im MRT übersehen werden und sollte immer auf der Grundlage der Ergebnisse der klinischen Untersuchung beurteilt werden.

Die MCL-Verletzungen werden im Allgemeinen in 3 Grade eingeteilt, wobei bei einer Verletzung des Grades III mit Valgusinstabilität in voller Streckung von einem hohen Risiko für eine gleichzeitige VKB-Verletzung und AMRI ausgegangen wird. Kürzlich schlugen Wierer et al. eine vereinfachte Klassifizierung der AMRI vor, die auf einer Kombination des anteromedialen Schubladentests, des externen Dial-Tests und des Valgusbelastungstestes basiert [49]. Die mediale Instabilität kann auch während der **Arthroskopie**

**Abb. 6:**
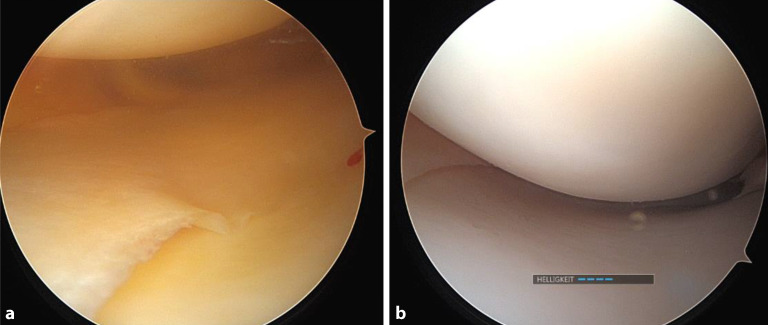
**a** Arthroskopischer Befund bei Ruptur des „deep medial collateral ligament“ (dMCL) und des „superficial medial collateral ligament“ (sMCL) mit daraus resultierend erweitertem medialen Gelenkspalt, **b** Befund nach Refixation

Arthroskopie

beurteilt werden: Unter Valgusbelastung kann ein **mediales Gapping**

mediales Gapping

(erweiterter Gelenkspalt) beobachtet werden (Abb. 6).

**Abb. 7:**
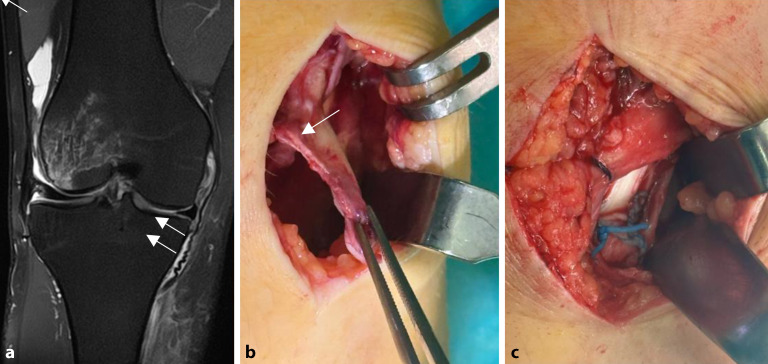
**a** Magnetresonanztomogramm mit Stener-like-Läsion (*Pfeile*) eines Skifahrers nach einem Valgustrauma; **b,** **c** intraoperativer Befund und Zustand nach Refixation mithilfe eines Nahtankers in anatomischer Position unterhalb des Pes anserinus

Ein Abheben des medialen Meniskus mit meniskofemoralem oder meniskotibialem Gapping kann beobachtet werden, was jeweils auf eine femorale oder tibiale dMCL-Ruptur hinweist. Auch eine AMRI kann durch eine Subluxation des Innenmeniskushinterhorns vor die mediale Kondyle unter Einleitung eines Außenrotationsstresses im Rahmen der Arthroskopie valide dargestellt werden. In akuten Fällen hat das MCL grundsätzlich ein gutes Heilungspotenzial, sodass eine Operation erwogen werden sollte, wenn keine ausreichende Heilung zu erwarten ist. Indikationen für eine Operation sind eine Valguslaxität des Grades 3 in voller Streckung und bei 30°, ein positiver Befund im Dial-Test für AMRI und eine MCL-Laxität des Grades 2 bei Sportlern in Verbindung mit einer VKB-Rekonstruktion. Eine weitere Indikation für eine Operation ist die „Stener-like“-Läsion (abgeleitet von der „Stener-Läsion“, des über die Aponeurose dislozierten gerissenen ulnaren Daumenseitenbands), eine tibiale Avulsion des sMCL, bei der der gerissene Bandanteil über den Pes anserinus verlagert wird (Abb. 7).

In der akuten Situation ist eine Reparatur der geschädigten Strukturen wünschenswert, z. B. mithilfe von **Nahtankern**

Nahtankern

zur Fixierung der ausgerissenen Strukturen. Wenn eine intratendinöse Läsion festgestellt wird, oder wenn nach der Reparatur eine Restlaxität verbleibt, kann eine **Augmentation**

Augmentation

mit einer flachen autogenen Grazilissehne durchgeführt werden [51].

In chronischen Fällen ist es notwendig, den anatomischen Zustand der beschädigten Strukturen durch Rekonstruktion mithilfe von Sehnentransplantaten wiederherzustellen [52]. Bei der Behandlung der medialen Instabilität in Verbindung mit VKB-Verletzungen ist die Verwendung der Semitendinosus-Sehne als VKB-Transplantat i. Allg. zu vermeiden, um eine Einschränkung der dynamischen Stabilisierung durch die HS zu vermeiden [53].

## Meniskusläsionen

Meniskusverletzungen sind häufige Begleitverletzungen von VKB-Rupturen. Die Inzidenz wird in der Literatur im akuten Stadium zwischen 55 und 85 % angegeben und kann bei chronischen Instabilitäten noch höher ausfallen [54].

Es ist von entscheidender Bedeutung, Meniskusrisse zeitnah (im Idealfall innerhalb weniger Wochen) zu behandeln, da diese mit der Zeit immer komplexer und weniger reparabel werden können. Insbesondere gilt dies auf der lateralen Seite. Außerdem erhöht eine Verzögerung der VKB-Rekonstruktion um mehr als 6 Monate das Risiko von **Sekundärläsionen**

Sekundärläsionen

oder eines Fortschreitens der ursprünglichen Läsionen (Meniskus/Knorpel; [55]). Die Behandlung von Meniskusläsionen hat sich in den letzten 30 Jahren weiterentwickelt. Da der Meniskus eine entscheidende biomechanische Rolle als sekundärer Stabilisator für die anteriore Translation und Rotationsstabilität spielt, gibt es eine zunehmende Tendenz, ihn zu erhalten. Trotzdem kann die **selektive Meniskektomie**

selektive Meniskektomie

eine Option sein, wenn die Erfolgsaussichten anderer **meniskusreparativer Techniken**

meniskusreparativer Techniken

gering eingestuft werden muss [56]. Häufig ist dies bei degenerativen Meniskusrissen, Rissen in der Weiß-Weiß-Zone, komplexen oder chronisch dislozierten Rupturen der Fall. In diesen Fällen sollte, wenn eine Meniskusektomie nicht vermieden werden kann, eine möglichst zurückhaltende Teilresektion durchgeführt werden. Besondere Aufmerksamkeit ist bei der Durchführung einer lateralen Meniskektomie geboten, da die Ergebnisse im Vergleich zur medialen Seite tendenziell schlechter sind. Dieser Unterschied ist auf anatomische und biomechanische Faktoren zurückzuführen: Der laterale Meniskus trägt einen größeren Teil der Belastung im lateralen Kompartiment als der mediale Meniskus im medialen Kompartiment (70 % gegenüber 50 %), aufgrund der geringeren Kongruenz des lateralen Kniekompartiments. Auch bei Profisportlern konnten nach partieller Resektion der Außenmenisken im Vergleich zum Innenmeniskus eine spätere Rückkehr zum Sport und eine höhere Wahrscheinlichkeit für Folgebeschwerden festgestellt werden [57, 58].

Die erfolgversprechendste Indikation für eine Meniskusreparatur ist eine instabile, vertikale oder longitudinale Läsion in der peripheren Region (rot-rote Zone) des Meniskus. Wichtig ist zusätzlich die Berücksichtigung allgemeiner Faktoren des Patienten wie Alter, Aktivitätsniveau, Kniestabilität und die Beinachse.

Die Erfolgsquote bei Meniskusreparaturen ist deutlich höher, wenn sie früh nach dem Trauma (< 12 Wochen) durchgeführt werden [54].

Verletzungen, die lange Zeit übersehen wurden, sind die **Rampenläsionen**

Rampenläsionen

(Risse der dorsalen meniskokapsulären Verbindung des Innenmeniskus). Deshalb sollte im Rahmen der VKB-Rekonstruktion routinemäßig die Visualisierung des Rampenbereichs mithilfe der Inspektion der dorsomedialen Kapselregion in „Gilchrist-View“ (Einbringen des Arthroskops durch den Interkondylarraum in den dorsomedialen Aspekt des Kniegelenks) durchgeführt werden. Größere Rampenläsionen sollten vorzugsweise über ein posteromediales Arbeitsportal direkt genäht werden. Große unbehandelte Rampenläsionen können zu einer verbleibenden anteroposterioren Instabilität in dem VKB-rekonstruierten Knie und zu schlechteren funktionellen Ergebnissen beitragen [59, 60].

## Fazit für die Praxis

- Eine möglichst der anatomischen Situation entsprechende Rekonstruktion des vorderen Kreuzbands (VKB) mithilfe individueller Transplantatwahl ist für ein gutes postoperative Ergebnis entscheidend.
- Eine Slope-Rekonstruktion sollte bei entsprechenden Werten vorrangig im Revisionsfall Anwendung finden.
- Die exakte Beurteilung der peripheren medialen und lateralen Strukturen sowie ihre entsprechende Therapie sind essenziell und sollten stets bedacht werden.
- Die Therapieplanung einer Meniskusverletzung hängt von der Läsionskonfiguration und anamnestischen Besonderheiten des Patienten ab.

## CME-Fragebogen

Welche Aussage zur Anatomie und zur Biomechanik des Kniegelenks ist korrekt?

Der tibiale Footprint des vorderen Kreuzbands zeigt sich dreiecksförmig.

Das vordere Kreuzband ist eine runde „kabelartige“ Struktur.

Femoral inseriert das native vordere Kreuzband über direkte und indirekte Fasern.

Das native Kreuzband ist etwa 30 mm breit und besteht aus 4 klassischen Bündeln.

In Extension fungiert das vordere Kreuzband als primärer Stabilisator der tibialen Außenrotation.

Sie planen eine Rekonstruktion des vorderen Kreuzbands ohne eindeutig sichtbaren Stumpf. Welche arthroskopische Landmarke dient Ihnen als Orientierung zur tibialen Tunnelanlage?

Vorderhorn des Außenmeniskus

Laterale Notch-Wand

Hinterhorn des Außenmeniskus

Vorderhorn des Innenmeniskus

Femoraler Ansatz des hinteren Kreuzbands

Die Transplantatwahl spielt bei der Rekonstruktion des vorderen Kreuzbands eine wichtige Rolle. Was gilt es, dabei zu beachten?

Ein „Bone-patellar-tendon-bone“(BPTB)-Transplantat stellt bei häufig knienden Tätigkeiten den Goldstandard dar.

Allogene Transplantate sind bezüglich der Versagensrate gleichwertig wie Autografts zu werten.

Quadrizepssehnentransplantate können sowohl mit als auch ohne Knochenblock verwendet werden.

Semitendinosussehnentransplantate eignen sich besonders für Sportler, die auf eine gut entwickelte Kniebeugemuskulatur angewiesen sind.

Biomechanisch besitzen allogene Transplantate bessere Struktureigenschaften.

Worauf sollte bei der Planung einer operativen Slope-Korrektur geachtet werden?

Nach derzeitiger Datenlage sollte diese nur im Revisionsfall durchgeführt werden.

Die supratuberositäre Osteotomie korrigiert eine Patella alta.

Die Indikation besteht ab einem posterioren tibialen Slope (PTS) > 5°.

Eine zu starke Korrektur kann zu einer Verstärkung der Hyperextension des Kniegelenks führen.

Die „Medial-closed-wedge“-Osteotomie stellt diesbezüglich ein gängiges Verfahren dar.

Welche Struktur ist wesentlich an der Aufrechterhaltung der anterolateralen Rotationsstabilität beteiligt?

Mediales patellofemorales Ligament (MPFL)

Tiefes mediales Kollateralband („deep medial collateral ligament“, dMCL)

Kaplan-Fasern, „anterolateral ligament“ (ALL), Kapsel

Medialer Meniskus

Hinteres Kreuzband

Welche Aussage zu Lateralen-extraartikulären-Tenodese(LET)-Verfahren im Rahmen der Rekonstruktion des vorderen Kreuzbands trifft zu?

Insbesondere profitieren davon Patienten ab einem Alter > 40 Jahren mit moderatem sportlichen Anspruch.

Eine LET sollte im Rahmen einer primären Ruptur des vorderen Kreuzbands nicht angewandt werden.

Das sog. Elmslie-Verfahren findet häufig Anwendung.

Durch eine LET kann die Rate der Rerupturen des vorderen Kreuzbands nicht reduziert werden.

Die Indikationsstellung ist abhängig von mehreren klinischen Merkmalen wie u. a. einem positiven Pivot-Shift-Test, der Hyperlaxität und der Revisionssituation.

Welche pathologische Veränderung des Meniskus sollte am ehesten mit einem Nahtverfahren versorgt werden?

Läsionen nach Trauma, > 6 Monate zurückliegend

Longitudinale Risse in der rot-roten Zone

Risse in der weiß-weißen-Zone

Komplexe Risse des medialen Meniskus

Chronisch dislozierte Rupturen

Ein Patient stellt sich nach einem Valgustrauma bei Ihnen vor. Welcher Testbefund lässt eine isolierte Verletzung des oberflächlichen medialen Kollateralbands („superficial medial collateral ligament“, sMCL) vermuten?

Positiver Dial-Test

Vermehrte anterolaterale Instabilität in 30°-Flexion

Vermehrte Aufklappbarkeit unter Valgusstress in voller Extension

Schmerzprovokation im lateralen Kompartiment

Vermehrte Aufklappbarkeit unter Valgusstress in 30°-Flexion

Wie sollte eine Verletzung des MCL(mediales Kollateralband)-Komplexes am ehesten therapiert werden?

Sogenannte Stener-Läsionen sollten aufgrund des guten Heilungspotenzials konservativ therapiert werden.

Falls nach operativer Refixation einer intratendinösen Ruptur eine Restlaxität verbleibt, sollte die Augmentation mittels Sehnentransplantat bedacht werden.

In der akuten Situation sollte eine Reparatur der geschädigten Strukturen nicht durchgeführt werden.

In chronischen Fällen erfolgt die direkte Refixation mittels Nahtankern.

Ein operatives Vorgehen ist bereits bei eingradiger isolierter Valgusinstabilität indiziert.

Welches intraoperative Vorgehen ist zum Beweis/Ausschluss einer „Rampenläsion“ indiziert?

Eine konventionelle Röntgenkontrolle

Eine mediale Arthrotomie

Eine arthroskopische Visualisierung der dorsomedialen Kapselregion mittels Gilchrist-View

Eine exakte Visualisierung ist erst nach subtotaler Meniskusentfernung möglich

Eine arthroskopische Visualisierung unter Anlage eines tiefen anteromedialen Portals
